# Relationship between *BRAF V600E* and *KRAS* mutations in stool for identifying colorectal cancer: A cross-sectional study

**DOI:** 10.1016/j.amsu.2020.10.027

**Published:** 2020-10-23

**Authors:** Warsinggih, Liliyanto, Marhamah, M. Ihwan Kusuma, Julianus Aboyaman Uwuratuw, Erwin Syarifuddin, Muhammad Faruk

**Affiliations:** a Division of Digestive, Department of Surgery, Faculty of Medicine, Hasanuddin University, Makassar, Indonesia; b Department of Pediatric Dentistry, Faculty of Dentistry, Hasanuddin University, Makassar, Indonesia; c Department of Surgery, Faculty of Medicine, Hasanuddin University, Makassar, Indonesia

**Keywords:** BRAF V600E, KRAS, Stool, Colorectal cancer

## Abstract

**Background:**

With early diagnosis, colorectal cancer (CRC) is a curable disease. As studies in the past 15 years have shown, specific genetic changes occur in the neoplastic transformation of normal colonic epithelium to benign adenoma until becoming adenocarcinoma. Considering that dynamic, we aimed to determine how v-raf murine sarcoma viral oncogene homolog B1 *(BRAF) V600E* and Kirsten rat sarcoma *(KRAS)* mutations relate to the location, histopathology, and degree of tumor differentiation in CRC.

**Methods:**

With a cross-sectional design involving an observational analytical approach, we determined the relationship of *BRAF V600E* and *KRAS* mutations to the location, histopathology, and degree of tumor differentiation in CRC.

**Result:**

The sample contained 43 patients with CRC aged 21–80 years, with an average age of 56.0 ± 11.2 years, 46.5% of whom were male and 53.5% female, for a male-to-female ratio of 1.0–1.15. Most tumors were located in the right colon (*n* = 18, 41.9%), followed by the rectum (*n* = 14, 32.6%) and left colon (*n* = 18, 25.6%). Non-mucinous adenocarcinoma was more prevalent than mucinous adenocarcinoma, with 22 (51.2%) and 21 (48.8%) patients, respectively. Nineteen tumors were poorly differentiated (44.2%), 15 were moderately differentiated (34.9%), and nine were well-differentiated (20.9%). *BRAF V600E* mutations totaled six (14%), whereas non-*BRAF V600E* mutations totaled 37 (86.0%). *BRAF V600E* mutations significantly related to tumor location, degree of differentiation, and histopathology (*p <* .01).

**Conclusion:**

A significant relationship exists between *BRAF V600E* mutations in the stool of patients with CRC and location, histopathology, and degree of tumor differentiation.

## Highlights

• Colorectal cancer (CRC) is the third-leading cause of cancer-related death worldwide.
• DNA fragment tests of stool can identify CRC.
• A significant relationship exists between *BRAF V600E* mutations in the stool of patients with CRC and location, histopathology, and degree of tumor differentiation.

## 1 Introduction

With early diagnosis, colorectal cancer (CRC) is a curable disease. As studies in the past 15 years have shown, specific genetic changes occur in the neoplastic transformation of normal colonic epithelium to benign adenoma until becoming adenocarcinoma [1]. Although CRC is the third-leading cause of cancer-related death worldwide [2,3], its mortality has been reduced due to early detection with population-based screening programs [4].

In Indonesia, the absence of population-based data has meant that no clear overview of the incidence of CRC there remains unavailable. In fact, various reports only show increases in the number of cases of CRC as one of the 10 most common cancers. Even so, data from the Indonesian Ministry of Health indicate an incidence of CRC totaling 1.8 people for every 100,000 in the population. In 2005, CRC ranked fourth highest among malignant cancers; however, by 2006, cases of CRC totaled 107, and the disease ranked third-most malignant, and by 2008, cases totaled 272, and malignancy had risen to second, following only breast cancer [5].

The prognosis of patients with CRC is heavily influenced by several factors, including clinical variables, stage, histopathology, and molecular Oncogenetic factors of the tumor. According to the literature, the discovery of *v-raf murine sarcoma viral oncogene homolog B1 (BRAF) V600E* mutations indicates the possibility of colon cancer with aggressive phenotypes, and such mutations have become a significant prognostic biomarker, especially in end-stage cases [6]. At the same time, *Kirsten rat sarcoma (KRAS)* mutations, typically analyzed in stool samples, are also prognostic factors in patients with CRC [7,8].

Given those findings and trends, in our study we sought to ascertain the relationship of *BRAF V600E* and *KRAS* mutations with the degree of differentiation in CRC.

## 2 Methods

To determine the relationship of *BRAF V600E* and *KRAS* mutations with the degree of differentiation in CRC, in our study we adopted a cross-sectional design guided by an observational analytical approach. Our research was conducted in a referral hospital in eastern Indonesia following the approval of the local ethics committee (no. 62/UN4.6.4.5.31/PP36/2019) and has been registered with the Research Registry (no. 5926). Herein, our work is reported in accordance with Strengthening the Reporting of Cohort Studies in Surgery (STROCSS) criteria [9].

### 2.1 Population and sample

Our study's population encompassed all patients diagnosed with CRC and treated at the referral hospital. We applied consecutive sampling to form the research sample in light of several inclusion criteria—patients had to have CRC before surgery, not exhibit total mechanical intestinal obstruction or perforation, and be willing to participate—and exclusion criteria, namely history of surgery, chemotherapy, or radiotherapy and incomplete clinical and histopathological data. We divided the patients into five age groups: less than 40 years old, 40–49 years old, 50–59 years old, 60–69 years old, and 70 years old or older.

### 2.2 Stool collection, processing, and storage

Fresh stool samples collected from the patients with CRC were immediately stored at temperatures of −20 °C and within 24 h transferred to environment of −80 °C for permanent storage.

### 2.3 DNA extraction

DNA extraction involved using Norgen Biotek Corp.‘s 27600 Stool DNA Isolation kit according to the manufacturer's protocol. The concentration of DNA in the sample was determined by the Nano Drop 2000 (100–200 mg), and the DNA was stored at −80 °C.

### 2.4 Polymerase chain reaction

Polymerase chain reaction (PCR) amplification and DNA sequencing were used to identify mutations in the most frequently mutated *BRAF* area: exon 11, codon 468; exon 15, codon 596; and exon 15, codon 600. Real-time PCR mutation assay did not reveal *KRAS* mutations in codons 12, 13, or 61. The Cat KT205 mini kit (Tiangen Biotech Co., Ltd., Beijing, China) for DNA PCR in stool was used to analyze *BRAF* and *KRAS* mutations following the manufacturer's instructions. Next, sequences were analyzed using Applied Biosystems 3700 DNA sequences (Thermo Fisher Scientific Inc. Massachusetts, USA) and mutations in the *BRAF* codon 600 were identified in the direct sequencing of exon 15 in the BRAFa 189-bp fragment in the exon *BRAF* 15 obtained using PCR. Nucleotide 1779 (i.e., thymidine) was converted to adenine with the BioEdit sequence alignment editor for the *BRAF V600E* mutation.

### 2.5 Data analysis

The obtained data were processed using Statistical package for the social sciences (SPSS) version 25 (IBM Corp. Released 2017. IBM SPSS Statistics for Windows, Version 25.0. Armonk, NY: IBM Corp.). The chi-square test was used to determine the relationship of genetic mutations in the sample.

## 3 Results

According to the presence of mutations in their stool samples, all 43 participants had CRC. Among the sample's other characteristics (Table 1), most patients were female (*n* = 23, 53.5%), while the most populous age group was 50–59 years (41.9%).

**Table 1:** Patient characteristics.

Variable		*N*	%
Sex	Male	20	46.5
	Female	23	53.5
Age (years)	<40	4	9.3
	40–49	6	14.0
	50–59	18	41.9
	60–69	10	23.3
	≥70	5	11.6
Differentiation	Poor	9	20.9
	Moderate	15	34.9
	Good	19	44.2
Location	Right colon	18	41.9
	Left colon	11	25.6
	Rectum	14	32.6
Histopathology	Mucinous	21	48.8
	Non-mucinous	22	51.2
*BRAF V600E* mutations?	Yes	6	14.0
	No	37	86.0
*KRAS* mutations?	Yes	0	0
	No	43	100

Regarding the distribution of CRC characteristics in the sample according to category of CRC differentiation, 44.2% of patients (*n* = 19) showed poor differentiation. Furthermore, by location, most CRC was in the right colon (*n* = 18, 41.9%). In all, 22 distributions (51.2%) related to histopathology.

**Fig. 1:**
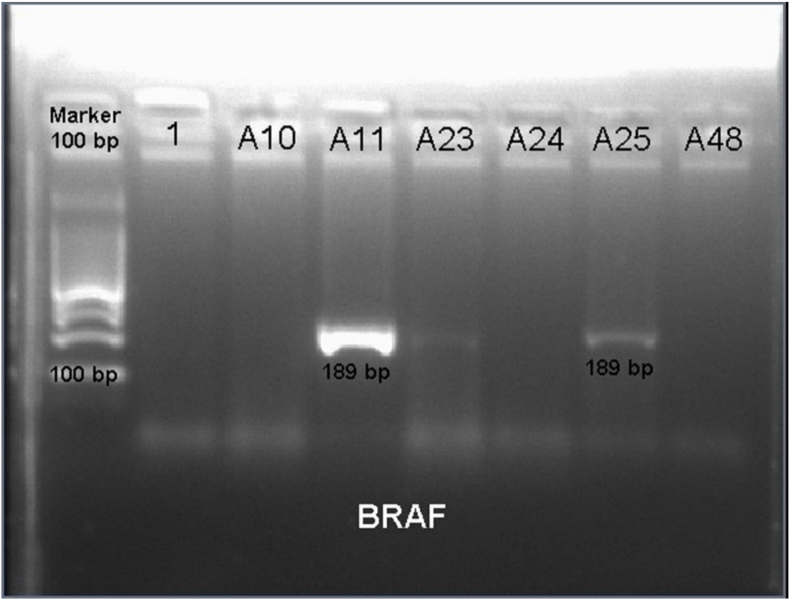
Positive polymerase chain reaction in the *BRAF* 189-bp fragment.

Most patients showed no change in color in the fluid around the location of the cancer. Stool sample examination conducted to determine the presence of genetic mutations in the occurrence of CRC revealed a mutation in *BRAF V600E*. Mutations in the *BRAF* codon 600 were identified with the direct sequencing of exon 15 in the *BRAF* 189-bp fragment (Fig. 1) in the exon *BRAF* 15 obtained using PCR. Nucleotide 1779 (thymidine) was converted to adenine, as shown in BioEdit for the *BRAF V600E* mutation. In all, six patients (14%) exhibited mutations.

By contrast, *KRAS* did not show any mutations in the sample. Among women, the percentage of *BRAF V600E* mutations was higher (83.3%), despite being higher among men without any mutations (51.4%). Even so, statistical tests revealed that the difference was not significant (*p >* .05). Meanwhile, the percentage of *BRAF V600E* mutations was highest for patients 60–69 years old (66.7%), and no mutations were found among ones less than 50 years old. Again, however, statistical tests revealed that the difference was not significant (*p >* .05), as shown in Table 2.

**Table 2:** The association of BRAF V600E mutation towards gender, and age.

		*BRAF V600E* mutation?		Total	p*
		Yes	No		
**Sex**					
Male	*n* (%)	1 (16.7)	19 (51.4)	20 (46.5)	.114
Female	*n* (%)	5 (83.3)	18 (48.6)	23 (53.5)	
**Age (years)**					
<40	*n* (%)	0 (0)	4 (10.8)	4 (9.3)	.075
40–49	*n* (%)	0 (0)	6 (16.2)	6 (14.0)	
50–59	*n* (%)	1 (16.7)	17 (45.9)	18 (41.9)	
60–69	*n* (%)	4 (66.7)	6 (16.2)	10 (23.3)	
≥70	*n* (%)	1 (16.7)	4 (10.8)	5 (11.6)	
**Location**					
Right colon	*n* (%)	6 (100)	12 (32.4)	18	.008
Left colon	*n* (%)	0	11 (29.7)	11	
Rectum	*n* (%)	0	14 (37.9)	14	
**Histopathology**					
Mucinous	*n* (%)	6 (100)	15 (40.5)	21 (48.8)	.009
Non-mucinous	*n* (%)	0 (0)	22 (59.5)	22 (51.2)	
**Degree of tumor differentiation**					
Poor	*n* (%)	6 (100)	13 (35.1)	19 (44.2)	.012
Moderate	*n* (%)	0 (0)	15 (40.5)	15 (34.9)	
Good	*n* (%)	0 (0)	9 (24.3)	9 (2.9)	

*Determined with the chi-square test.

Also shown in Table 2, a significant association arose between *BRAF V600E* mutations and tumor location (*p* < .01), and all *BRAF* mutations occurred in the right colon. We also detected a significant association between *BRAF V600E* mutations and degree of tumor differentiation (*p <* .05). Although all *BRAF* mutations were poorly differentiated, a significant association also emerged between *BRAF V600E* mutations and tumor histopathology (*p <* .01). On top of that, *BRAF* mutations surfaced in all mucinous adenocarcinoma. Our study additionally revealed that a higher percentage of *BRAF V600E* mutations among females (83.3%) than males (51.4%), although the difference was not significant (*p >* .05). The percentage of *BRAF V600E* mutations in people 60–69 years old was 66.7%. Last, despite mutations in patients less than 50 years old, the difference was not significant (*p >* .05).

## 4 Discussion

Because CRC involves non-specific symptoms with long-term intervals associated with the initial appearance of gland metastases [10,11], few patients diagnosed with the disease receive curative surgery. At the same time, because CRC is believed to develop slowly via the progressive accumulation of genetic mutations, the risk of recurrence and death due to CRC is strongly associated with the disease's stage at diagnosis. Early disease detection and interventions performed at that stage can reduce the risk of death due to CRC [[10], [11], [12], [13], [14]].

Age is a dominant risk factor in CRC. As previously reported, the risk of developing CRC increases with age, especially among both men and women who are at least 50 years old, and only 3% of CRC occurs in people under the age of 40 years [15,16]. We determined a similar finding: that the most common age group to have CRC was 50- to 59-year-olds. Research has shown that CRC detected in younger patients is usually advanced and marked by acute differentiation [17]. Offering additional support, an increase in CRC toward all colonic sites in older adults was previously found in an analysis of metastasized CRC [18].

Our findings also include that *BRAF V600E* mutations were more common in women than men. Men were more likely to experience somatic *BRAF* mutations, with an effect that was stronger in smokers with tumors showing high microsatellite instability (MSI) [19]. On that topic, Lochead et al. (2013) found that BRAF–MSI mutations tend to affect older women and be located proximally [20]. The highest percentage of *BRAF V600E* mutations occurred in patients in their 70s, whereas no mutations were observed in ones less than 50 years old (Table 2). However, those differences were not significant (*p >* .05).

Our study additionally showed that gene mutation was higher in women than men, particularly regarding the site of the *BRAF* mutation. That finding is consistent with the results of Tsai et al. (2018), who demonstrated that women developed more *BRAF* gene mutations than men (6.4 vs. 3.3%, OR = 1.985, *p* = .006) [21].

We also found a significant relationship between *BRAF V600E* mutations and tumor location (*p <* .01), as detailed in Table 2. A strong correlation additionally arose between *BRAF V600E* mutation and tumor location. In that light, our findings are comparable to White et al.‘s (2018), which revealed that the proportion of CRC in the right colon was more common in women (27%) than men (19.5%)] [22]. Kalady et al. (2012) similarly found that *BRAF* mutations were rare in the left colon, absent in the rectum, and primarily located in the proximal colon (44% vs. 95%, *p <* .001) [23]. Added to those findings, Tie et al. (2011) observed that *BRAF* mutations were common in the right colon (22%, 41/147, *p <* .0001) but rare in the left colon (4%, 7/153) and rectum (2%, 4/173) [24]. Domingo et al. (2004) reported that tumors in the proximal colon were 4.76-fold more likely to have a *BRAF V600E* mutation than ones in the distal or rectal colon [25].

The most common histopathological finding in our study was the mucinous type versus the non-mucinous type. In particular, we found a significant relationship between *BRAF V600E* mutations and histopathological features (*p <* .01) for mucinous tumors (Table 2). CRC can develop alongside several histological patterns, often distinguished by the degree to which goblet cells produce and secrete mucin. Of all CRCs, the mucinous and signet ring cell types are rare (10%–20%) and associated with worse survival rates [26]. Pai et al. (2012) found that *BRAF* mutations were also high in the histological type of mucinous, serrated, poorly differentiated CRC with high MSI. They also reported that BRAF–microsatellite stable (MSS) mutations are similar to *BRAF* microsatellite instable (MSI) mutation patterns in mucinous and poorly differentiated CRC [27].

Mucinous cancer differs from CRC found in 3.9%–19% of people with the disease. In particular, mucinous cancer differs from adenocarcinoma in terms of clinical and histopathological characteristics [26] and is more common in patients with colon cancer than rectal cancer (15% and 9%, respectively). A subtype of CRC more common in women than men, it also tends to be located on the proximal side of the colon. Despite such findings, the etiology of mucinous cancer remains uncertain [28].

Mutations were more common among patients with mucinous CRC than non-mucinous carcinomas. In turn, that poor prognosis relates to the histology of mucinous disease and may be partly explained by mutations in the *BRAF* gene. Those specific clinical features support the hypothesis that BRAF-mediated carcinogenesis in CRC commences with changes in the function of the *BRAF* gene as an initial step in the serrated pathway, which ultimately prompts RAF–MEK–ERK–MAP signaling activity [29].

We also found a significant relationship between *BRAF V600E* mutations and the degree of tumor differentiation (*p <* .05), as detailed in Table 2. Jang et al. (2017) evaluated clinicopathological characteristics of CRC in relation to *BRAF V600E* mutations a subgroup of patients with MSS or MSI. In that subgroup, CRC with *BRAF V600E* mutations was more likely mucinous peritoneal carcinomatosis in a proximal location at an advanced pT stage and with larger tumors, a serrated component, poorly differentiated histology, and lymph vascular invasion than other types of CRC [30]. Roth et al. also concluded that *BRAF* mutations significant relate to femaleness, right colon location, older age, advanced stage of TNM classification, tumors with high MSI, mucinous histology, high the CpG island methylator phenotype (CIMP) status, and the MutL homolog 1 (MLH1) and that such mutations prompted poor differentiation and a poor prognosis [31,32].

A limitation of our study was the limited number of patients in the sample due to time and cost constraints. Equipment for testing DNA fragments is expensive, while obtaining the results of *BRAF V600E* and/or *KRAS* mutations in the stool of CRC patients is time-consuming.

CRC with *KRAS* mutations can arise through the serrated and chromosomal instability (CIN) pathways [33,34]. Patai et al. have postulated that the *KRAS* serrated mutation pathway activates the mitogen activated protein kinase (MAPK) pathway, meaning that it causes no apoptosis, which induces serrated histomorphological features as a result of cell proliferation. Although KRAS mutations have occurred in 30–50% of cases of CRC [33], we found no mutation in KRAS. In fact, when a correlation surfaced between CRC and expressed KRAS mutations, then no clinical variables were significantly related. That finding aligns with the theory articulated by Murcia et al. [35].

## 5 Conclusion

A significant relationship exists between *BRAF V600E* mutations in the stool of patients with CRC and the tumor location, histopathology, and degree of tumor differentiation. Our research thus shows that testing DNA fragments in stool can identify CRC.

## Funding

This study was supported by funds from the Ministry of Research and Technology of the Republic of Indonesia through a research grant for novice researchers entitled “RISTEKDIKTI” Number: 641/UN4.1/KEP/2019.

## Ethical Approval

All procedure for human experiment has been approved by Ethics Commission Faculty of Medicine, Hasanuddin University Number: 62/UN4.6.4.5.31/PP36/2019.

## Consent

The research was conducted ethically in accordance with the World Medical Association Declaration of Helsinki. The patients have given their written informed consent on admission to use their prospective data base and files for research work.

## Author contribution

WS, LY, MIK, JAB, and ES wrote the manuscript and participated in the study design. WS, WS, LY, MIK JAB, ER, and MF drafted and revised the manuscript. WS, LY, MIK, JAB, and ER performed treatment and surgery. WS, LY, MH, and MF performed bioinformatics analyses and revised the manuscript. All authors read and approved the final manuscript.

## Registration of Research Studies

This study is registered with the Research Registry and the unique identifying number is: researchregistry5926.

## Guarantor

Warsinggih.

## Declaration of competing interest

The authors declare that they have no conflicts of interests.
